# Early-life gut microbiota and neurodevelopment in preterm infants: a narrative review

**DOI:** 10.3389/fnut.2023.1241303

**Published:** 2023-08-08

**Authors:** Isadora Beghetti, Monica Barone, Patrizia Brigidi, Alessandra Sansavini, Luigi Corvaglia, Arianna Aceti, Silvia Turroni

**Affiliations:** ^1^Neonatal Intensive Care Unit, IRCCS Azienda Ospedaliero-Universitaria di Bologna, Bologna, Italy; ^2^Department of Medical and Surgical Sciences, University of Bologna, Bologna, Italy; ^3^Microbiomics Unit, Department of Medical and Surgical Sciences, University of Bologna, Bologna, Italy; ^4^Department of Psychology “Renzo Canestrari”, University of Bologna, Bologna, Italy; ^5^Unit of Microbiome Science and Biotechnology, Department of Pharmacy and Biotechnology, University of Bologna, Bologna, Italy

**Keywords:** preterm infants, gut microbiota, *Bifidobacterium*, gut-brain axis, neurodevelopment, brain maturation, developmental windows

## Abstract

Infants born preterm are at a high risk of both gut microbiota (GM) dysbiosis and neurodevelopmental impairment. While the link between early dysbiosis and short-term clinical outcomes is well established, the relationship with long-term infant health has only recently gained interest. Notably, there is a significant overlap in the developmental windows of GM and the nervous system in early life. The connection between GM and neurodevelopment was first described in animal models, but over the last decade a growing body of research has also identified GM features as one of the potential mediators for human neurodevelopmental and neuropsychiatric disorders. In this narrative review, we provide an overview of the developing GM in early life and its prospective relationship with neurodevelopment, with a focus on preterm infants. Animal models have provided evidence for emerging pathways linking early-life GM with brain development. Furthermore, a relationship between both dynamic patterns and static features of the GM during preterm infants’ early life and brain maturation, as well as neurodevelopmental outcomes in early childhood, was documented. Future human studies in larger cohorts, integrated with studies on animal models, may provide additional evidence and help to identify predictive biomarkers and potential therapeutic targets for healthy neurodevelopment in preterm infants.

## 1. Introduction

Over the last two decades, the impact of the gut microbiota (GM) on host health and physiological processes, including neurodevelopment, has been the subject of increasing research (1–4). However, only few studies have explored the relationship between GM assembly, brain growth, and neurodevelopment in preterm infants (5–11). As a result of continuous improvements in neonatal intensive care, the mortality rate of extremely preterm infants [i.e., those with a gestational age (GA) of less than 28 weeks] has dramatically decreased over time. However, the improved survival of these infants is associated with a substantially elevated risk of severe morbidities and life-long neurodevelopmental impairment (including cerebral palsy, autism-spectrum disorders, anxiety, antisocial behaviors, and learning disabilities) (12). The third trimester of pregnancy is a critical period for brain growth and function, during which the brain increases significantly in volume, and cognitive function gains complexity (13, 14). Preterm birth interrupts the physiological growth and development of the brain that would have occurred during the third trimester of pregnancy. Furthermore, preterm brain development is hampered postnatally by a variety of noxious environmental stimuli and insults that are closely linked to neonatal immaturity and the neonatal intensive care unit (NICU) environment (15), including early microbial colonization. Accumulating evidence suggests that, during early-life, GM is involved in bidirectional signaling between the gut and the brain, forming the so-called microbiota-gut-brain axis (MGBA) (4). However, in premature infants, the GM-host relationship is likely to be severely impaired, predisposing preterm infants to adverse outcomes, such as necrotizing enterocolitis (NEC) and late-onset sepsis (LOS), ultimately interfering with the MGBA (16).

In this narrative review, we first provide an overview of the developing GM in early life, then discuss the emerging pathways linking GM and brain development, including current animal models, and the potential prospective relationship with neurodevelopment. Finally, we aim to provide an up-to-date review of available studies that have specifically explored the relationship between early-life GM and neurodevelopmental outcomes in preterm infants.

## 2. Gut microbiota in early life: assembly and influencers

Newborns born at term, vaginally, exclusively breastfed and not exposed to antibiotics have the ideal characteristics of a healthy early-life GM (17). One of the most important factors influencing microbial colonization patterns in newborns is the vertical transmission of bacteria from mother to child (18). At the time of birth, the passage of the baby through the birth canal represents the first event of exposure, first to microbes present in the vagina, on the mother’s skin and in feces, and subsequently to microbes present in the surrounding environment (19). This event represents early maternal imprinting, which plays a pivotal role in the assembly and maturation of the GM in early childhood. Consequently, any event potentially capable of preventing the vertical transmission of the mother microbiota may potentially alter the primary colonization in the newborn. The assembly of the GM is also influenced by the mode of delivery. In particular, Cesarean delivery has an enormous perturbing influence in the context of term deliveries during the perinatal period (20–22), even independent of antibiotic exposure (22–25). The early colonizer community in Cesarean-born infants borders in composition on the microbial community associated with the mother’s skin, as well as that present in the operating room, and is characterized by a depletion of Bacteroidetes compared to vaginally delivered infants (24, 26). Disruption of maternal microbiota transmission has been associated with a greater representation of opportunistic pathogens, even those resistant to antimicrobials, which is a risk factor for compromising neonatal health (23, 27). During early development, any disruption of GM-host interactions could irreversibly damage the infant priming process, thus hindering the establishment of a healthy homeostasis, and the existence of a critical period has been proposed (24, 28). Such disruptions are a major contributor to developmental issues, predisposing infants to develop impaired intestinal barrier function, inflammatory and metabolic diseases (29, 30), as well as alterations in communication with the brain *via* the MGBA, reflected in an increased risk of developing neurological diseases (31).

## 3. Emerging pathways linking gut microbiota to brain development: lessons from animal models

Accumulating evidence suggests that GM plays a role in several aspects of the host central nervous system (from development to function) through direct and indirect communication with the brain along the MGBA (32–34). However, the underlying mechanisms are far from being fully elucidated. Below, we discuss the emerging pathways linking GM to healthy or impaired neurodevelopment, and the major microbial intermediates involved [i.e., short-chain fatty acids (SCFAs), histamine, and tryptophan derivatives].

Such information has been derived from murine models [including germ-free (GF) mice, specific pathogen-free (SPF) mice, and other specific models], which, despite obvious limitations mainly due to differences in brain structure and physiology compared to humans, provide a powerful tool for mechanistic insights. Over the past 10 years, behavioral and cognitive assessments in juvenile GF mice have demonstrated the potential role of GM in influencing host neurodevelopment (35). Similarly, the comparative evaluation of motor activity and anxiety-related behaviors in GF mice vs. SPF mice allowed the researchers to highlight the potential involvement of intestinal microorganisms in the MGBA (36). In particular, GF mice showed increased motor activity and decreased anxiety, suggesting that microbial colonization may be an integral part of brain developmental programming, initiating signaling mechanisms that influence neuronal circuits related to motor control and anxiety-like behavior. Regarding the impact on brain maturation in early life, in a recent study, Lu et al., evaluated the effects on postnatal brain development in GF mice colonized with the GM of preterm infants known to induce high- or low-rate growth phenotypes (37). The GM configuration associated with the stunted phenotype was linked to an increase in neuroinflammation and a decrease in circulating insulin-like growth factor-1 (IGF-1), suggesting an unfavorable impact of particularly dysbiotic GM layouts on the early development of neurons and oligodendrocytes (37). In addition, Zhou et al. (38) demonstrated in a murine model of NEC that the presence of gut-released interferon-γ-producing CD4+ T cells in mice was associated with features of brain injury that are also observed in human infants with NEC, such as microglial activation, inflammation, and myelin loss (39, 40).

Several studies have also demonstrated impaired working memory functioning in GF mice related to decreased hippocampal levels of 5-hydroxytryptamine receptor 1A (5-HT1A) and brain-derived neurotrophic factor (BDNF) (41, 42). Increases in dopamine, serotonin (5-HT), and synaptic vesicle proteins were also observed in the striatum of GF mice, affecting motor and emotional responses in a brain region closely related to the basal ganglia and motor limbic, and causing anxiety-like behavior (36). In addition, lower levels of N-methyl-D-aspartic acid receptor (NMDAR), 5-HT1 receptor, and BDNF were found in the amygdala, which is part of the “emotional brain” limbic system, leading to increased risk-taking behavior (41, 43). Finally, GF mice exhibited an exaggerated hypothalamic–pituitary–adrenal (HPA) stress response, suggesting that the presence of GM from early developmental stages is required for the HPA system to become fully susceptible to inhibitory neural regulation (43).

Interestingly, a differential role for host genetics and GM features on neurodevelopmental outcomes has been documented in a specific mouse model, Cntnap2^−/−^, in which the hyperactive phenotype was linked to host genetics, whereas the social behavior phenotype was mediated by GM features (44). In this murine model, social deficits were restored by specific microbial interventions (i.e., administration of *Lactobacillus reuteri*), with the upregulation of metabolites involved in the synthesis pathway of tetrahydrobiopterin, a coenzyme relevant for the alleviation of symptoms related to social behavior in individuals with autism spectrum disorders (45). Finally, the maternal immune activation (MIA) murine model allowed the identification of potential probiotic therapies to alleviate gastrointestinal and behavioral symptoms associated with neurodevelopmental disorders (3). Specifically, Hsiao et al., demonstrated that administration of the human commensal *Bacteroides fragilis* to MIA offspring altered GM composition, positively modulated intestinal permeability, and ameliorated specific behaviors associated with autism spectrum disorders (3).

The MGBA is composed of several bidirectional pathways, involving neural, hormonal, and immunological signaling (46). Several microbial metabolites, such as SCFAs, histamine, and tryptophan derivatives, are essential mediators along this axis (47–50). SCFAs (derived from microbial fermentation of complex polysaccharides) play a pivotal role in promoting the maturation and proper functioning of microglia (51), which is in turn involved in early neurodevelopment and is responsible for antigen presentation, phagocytosis, and inflammatory regulation (52, 53). *In vitro* tests on organotypic slice cultures also showed that butyrate may act directly on oligodendrocytes to suppress demyelination, enhance remyelination, and promote oligodendrocyte differentiation, all critical factors in the pathogenesis of multiple sclerosis (54). Murine models deficient in the SCFA receptor FFAR2 exhibited microglial defects commonly associated with GF conditions, such as alterations in cell number and phenotype, resulting in an impaired innate immune response (51). Histamine, primarily produced in the gastrointestinal tract by *Escherichia coli* and *Morganella morganii* (55), is also important for microglial signaling involved in the regulation of host behavior and cognition, and contributes to microglia-mediated inflammation in the brain (56, 57). Finally, an important role in the regulation of MGBA has been hypothesized for tryptophan derivatives of GM origin. These microbial metabolites have the potential to affect neuroinflammation, nerve signal transduction, and blood–brain barrier maintenance by activating aryl hydrocarbon receptors on astrocytes and microglia, resulting in an overall suppression of inflammation (58). Derived from 5-hydroxytryptophan, serotonin is produced by several clostridial species (49) and also plays a key role in neurodevelopment, influencing neuronal differentiation and migration, axon growth, myelination, and synaptogenesis (46, 60).

## 4. The case study of preterm infants

### 4.1. Gut microbiota in preterm infants

The structural and immunological immaturity of the gut, which is distinctive of preterm infants, coupled with specific environmental conditions (delivery mode, NICU procedures and environment, drug administration, feeding), can severely interfere with a healthy microbial colonization (61). Indeed, lower GM diversity, wide inter-individual variation and increased proportions of potential pathogens are typically observed. For example, antibiotic exposure is known to reduce GM diversity and influence its composition, with an overabundance of Proteobacteria, to the detriment of Clostridia and *Bifidobacterium* (62). Colonization by the latter microbial genus is delayed and much less abundant in preterm than in term infants (63). The type of feeding has also a strong influence on preterm GM (64). Mother’s own milk feeding, compared to donor human milk and formula, induces higher GM diversity (65, 66) and *Bifidobacterium* abundance (67), potentially mitigating the detrimental effect of low birth weight/low GA.

The role of other microorganisms, such as fungi and archaea, that can colonize the infant gastrointestinal tract, is far from being fully understood (68–70), but the need to explore the inter-kingdom interactions that influence the assembly and maturation dynamics of the GM ecosystem is recognized. In a landmark study, Rao and colleagues have delved into the interplay between different kingdoms and showed that a single fungal species–*Candida albicans*–inhibited several dominant gut bacterial genera (71). The authors highlighted the centrality of simple microbe-microbe interactions in shaping the host-associated microbiota, which is critical for fully exploiting potential microbiota-based solutions to address altered microbiota configurations as well as impaired brain maturation and health outcomes in preterm infants.

### 4.2. Microbiota-gut-brain axis and signaling in preterm infants

**Figure 1:**
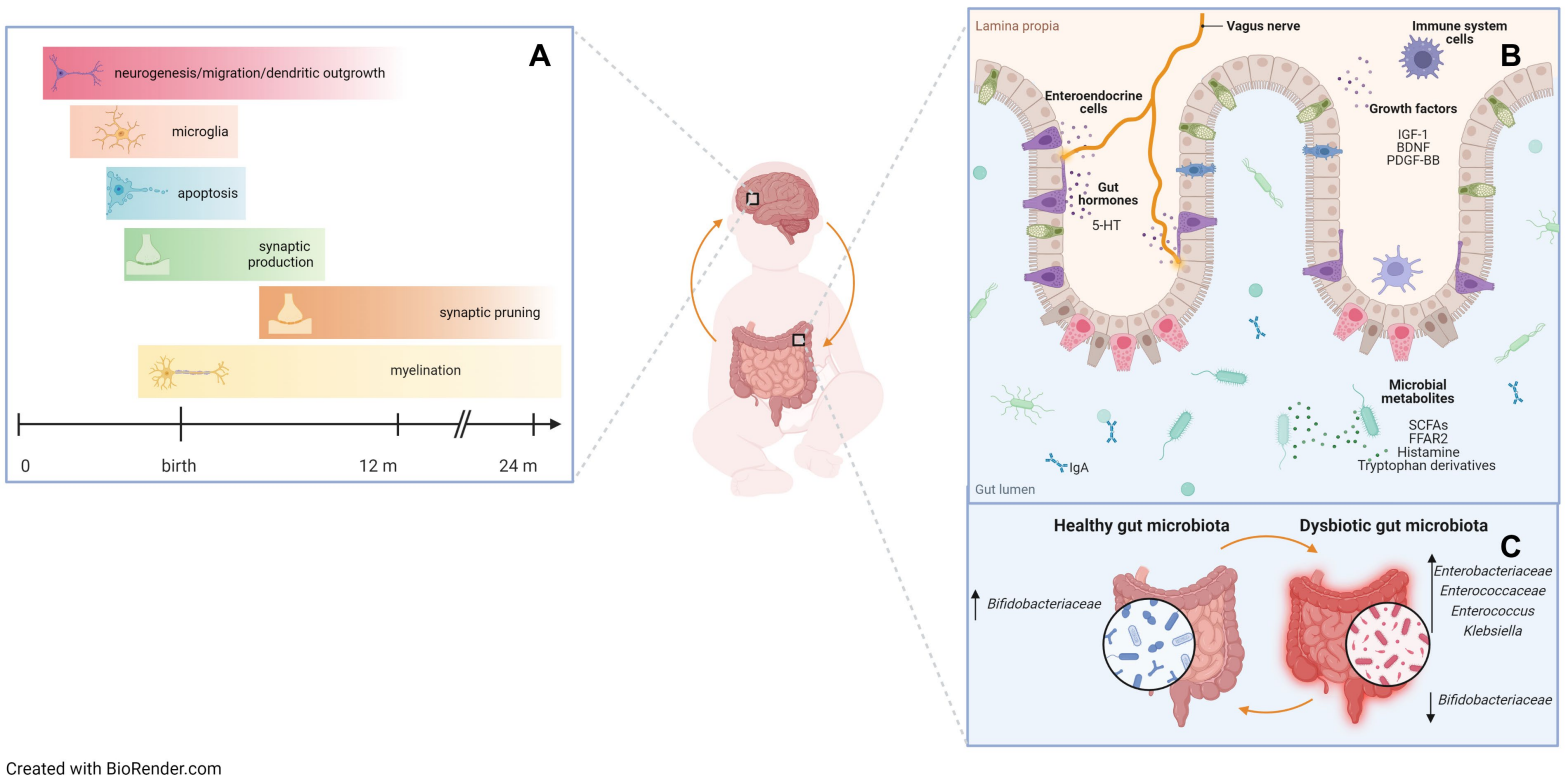
A critical window in early life for gut microbiota assembly and neurodevelopment. Preterm infants are at high risk of both gastrointestinal and neurodevelopmental impairment due to a peculiar developmental environment, with impaired gut microbiota assembly. **(A)** Brain developmental events during prenatal and early postnatal life that correspond to windows of vulnerability. Developmental processes occur in phases, setting the stage for potential periods of susceptibility to stimuli and insults that may affect brain growth and function. **(B)** Bidirectional gut-brain communication pathways. Evidence from animal studies suggests that gut hormones, growth factors, microbial metabolites, and receptors are involved in the microbiota-gut-brain axis. 5-HT: 5-hydroxytryptamine or serotonin; IGF-1: insulin-like growth factor-1; BDNF: brain-derived neurotrophic factor; PDGF-BB: platelet-derived growth factor-BB; SCFAs: short-chain fatty acids; FFAR2: free fatty acids receptor 2. **(C)** Dysbiotic gut microbiota profiles negatively affect gut-brain communication. Some specific bacterial taxa have been shown to be associated with neurodevelopmental outcomes in preterm infants. Up arrows indicate an increase in relative abundance of taxa, down arrows indicate a decrease in relative abundance of taxa.

Brain development begins *in utero* during the first month of pregnancy and involves a predefined sequence of events, many of which continue into postnatal life (72). Shortly before birth, approximately half of all neurons are cleared through apoptosis, with a second wave of synaptic pruning and elimination occurring during the peri-adolescent period (73). Numerous windows of vulnerability have been identified during prenatal and postnatal brain development. Within these windows, adverse events can significantly alter developmental trajectories and increase the risk of disease (74). For these reasons, infants born prematurely at the verge of the second and third trimesters represent a particularly vulnerable population (Figure 1), as they are at increased risk of perinatal white matter injury (PWMI), which may present with intraventricular hemorrhage, periventricular leukomalacia, or diffuse white matter injury (75). Perinatal inflammation and infections have been implicated in the pathogenesis of PWMI and may further worsen the neurological outcome (39). Interestingly, NEC, which is featured by GM dysbiosis (i.e., increased Proteobacteria levels and Toll-like receptor 4 activity) (76, 77) is associated with a significant risk of neurodevelopmental impairment (78, 79). Studies modeling neonatal infections have described the characteristics of neuroinflammation and documented the production of proinflammatory cytokines in the brain similar to those observed in the gut (40).

Peculiar GM compositions in the first months of life have also been associated with later neurodevelopmental outcomes. For example, Carlson et al. first performed GM 16S rRNA gene sequencing in 89 healthy term infants at 1 year of age and correlated GM features with Mullen Scale of Early Learning (MSEL) scores and brain imaging at 1 and 2 years of age (80). The authors grouped infants according to GM features at 1 year of age: infants in the GM group characterized by a high abundance of Bacteroidetes had better MSEL scores at 2 years of age, especially in receptive and expressive language, with breastfeeding and vaginal birth acting as covariates predicting a better outcome. One-year GM alpha diversity was inversely correlated with 2-year MSEL score. On the other hand, minimal effects of 1-year GM features on brain volume at 2 years of age were found.

More recently, in a study on 309 healthy term infants exploring the relationship between GM at 3–6 months of age and the Age and Stage Questionnaire (ASQ) score at 3 years of age (81), the authors documented specific associations between early GM composition and neurodevelopmental outcomes: infants with a high abundance of *Lachnospiraceae* and Clostridiales and a low abundance of Bacteroidetes in their GM performed worse in communication and personal social skills, while infants with an early GM dominated by Bacteroidetes and low in *E. coli* and *Bifidobacterium* had lower fine motor skill scores.

Aatinski et al., investigated the relationship between GM composition in 301 infants, aged 2.5 months, from the FinnBrain Birth Cohort Study, and infant temperamental traits, by administering the Infant Behavior Questionnaire-Revised (IBQ-R) 6 months after birth (82). The composition of the GM was grouped into three different community types, each characterized by specific microbial features. For example, infants in the high *Bifidobacterium*/*Enterobacteriaceae* abundance group scored the highest on the regulation trait, while infants in the *Bacteroides* group scored the lowest. In addition, some temperamental traits were associated with GM diversity and genus-level composition, even after adjusting for potential confounders such as mode of delivery and breastfeeding.

Given the early-life window of vulnerability of preterm infants for both GM assembly and neurodevelopment, recent studies, summarized in Table 1, have explored the relationship between early-life GM layout and neurodevelopmental outcomes in this specific population.

**Table 1:** Human studies exploring the relationship between early life gut microbiota and neurodevelopment outcomes in preterm infants.

Author, year (Reference)	Study details	Study population	Intervention	Gut microbiota assessment timing method	Neurodevelopment assessment timing method	Results
Beghetti et al. (2021) Italy (6)	O P M	Preterm infants <32 weeks GA [*n* = 27, median GA 30.6 (IQR 28.6–33.6) weeks]	NA	1, 4, 7, and 30 days of life 16S rRNA Illumina sequencing	24-month CA Griffiths Mental Development Scale (GMDS-R) and General Development Quotient (GQ) performed by psychologist	Early-life GM of infants with normal vs. impaired neurodevelopment followed distinct temporal trajectories with peculiar compositional rearrangements. Early *Bifidobacterium* deficiency appeared to be a negative biomarker f adverse neurological outcomes.
Oliphant et al. (2021) USA (8)	O P M	Preterm infants <34 weeks GA (*n* = 58)	NA	Weekly during NICU hospitalization until discharge or 36 weeks PMA 16S rRNA Illumina sequencing	Head Circumference Growth (HCG) weekly during NICU hospitalization until discharge or 36 weeks PMA	Preterm infants with suboptimal HCG trajectories had a depletion in the abundance/prevalence of Bacteroidota and *Lachnospiraceae*, independent of morbidity and caloric restriction.
Rozé et al. (2020) France (5)	C P Multic.	Preterm newborns born at 24 to 31 weeks GA [*n* = 577, mean GA 28.3 (SD 2.0) weeks]	NA	Week 4 after birth 16S rRNA Illumina sequencing	2 years CA Survey assessing cerebral palsy completed by the referring physician and parent assessed 24-month Ages and Stages questionnaire (ASQ)	GM cluster driven by *Enterococcus* and cluster driven by *Staphyloccoccus*, were significantly associated with 2-year non optimal outcome.
Sarkar et al. (2022) United States (7)	O P M	Preterm infants with birth weight < 1,500 g [*n* = 24, mean GA 27.95 (SD 1.81) weeks]	NA	Weekly for 6 weeks after NICU admission and at 2 and 4 years of age	2 and 4 years of age Battelle Development Inventory-2Screening Test (BDI-2ST) administered by researcher team scored by psychologist	Both NICU infant stool diversity and particular microbial ASVs were associated with BDI-2 ST cognition, adaptive, and communication subscales. Network analysis of the NICU infant stool microbial ecology showed differences in children needing neurodevelopmental referral.
Seki et al. (2021) Austria (9)	O P M	Extremely preterm infants [*n* = 60, mean GA 25.5 (SD 1.2) weeks]	NA	Days 3, 7, and 14, followed by biweekly sampling until discharge	Brain injuries identification by cUS and neurophysiological development assessment by aEEG (days 3, 7, and 14, then biweekly until discharge); cMRI at term-equivalent age	*Klebsiella* overgrowth in the gut was highly predictive for brain damage and was associated with a pro-inflammatory immunological tone.
Sun et al. (2020) United States (10)	O P M	Preterm infants [*n* = 34, mean BW 1451. (SD 479.3) g]	NA	Daily from 5 to 28 days of life 16S rRNA Illumina sequencing	36–38 weeks of post-menstrual age or prior to hospital discharge NICU Network Neurobehavioral Scale (NNNS)	A functional log-contrast regression model identified microbiota components at order (Clostridiales, Lactobacillales, Enterobacteriales) and genus level (*Veillonella*, *Enteroccoccus*, *Shigella*) that were associated with the neurobehavioral outcome of infant assessed by Stress/Abstinence subscale (NSTRESS)
Van den Berg et al. (2016) Netherlands (11)	RCT DB M	Very preterm infants GA < 32 weeks and/or BW < 1,500 g [*n* = 77 mean GA 29.9 (SD 1.7) weeks]	scGOS/lcFOS/pAOS or placebo supplemented to breast milk or to preterm formula days 3–30 of life	days 1, 7, 14 and 30 fluorescent *in situ* hybridisation (FISH) analysis	24 months CA Bayley Scales of Infant and Toddler Development (BSID) administered by blinded psychologist	Lower percentages of bifidobacteria at days 7 and 14 were associated with lower mental developmental index. Total bacterial count did not influence mental and psychological developmental index scores.

RCT, randomized controlled trial; P, prospective; B, blinded; DB, double-blinded; C, cohort; O, observational; M, monocentric; Multic, multicentric. BW, birth weight; GA, gestational age; PMA, postmenstrual age; GM, gut microbiota; ASVs, amplicon sequence variants. cUS, cranial ultrasound; aEEG, amplitude-integrated electroencephalography; cMRI, cranial magnetic resonance imaging. NA, not applicable.

Seki et al., described the relationship between the microbiota-immune-gut-brain axis and early neurodevelopment in 60 extremely preterm (GA < 28 weeks) and extremely low birth weight (BW < 1,000 g) infants (9). The authors described the characteristics of brain development over time in early life, assessed at multiple timepoints by cranial ultrasound and amplitude-integrated electroencephalography (aEEG) and at term-equivalent age by cerebral MRI, and identified a number of potential biomarkers of brain damage in this vulnerable population, including specific features of GM and immune function. Specifically, three distinct stages of brain development, from birth to term-equivalent age, were detailed in extremely preterm infants: first a quiescent phase, followed by a period of neurophysiological maturation, and then a term-equivalent phase. In infants with PWMI, specific microbial and immune features during the quiescent phase can trigger an inflammatory cascade, characterized by T-cell polarization and secretion of proinflammatory cytokines. Inflammation continues during the neurophysiological maturation period, which has a delayed onset and specific pathological features, such as alterations in brain electrical activity, cranial oxygen saturation, and neuroprotective secretion (i.e., platelet-derived growth factor-BB [PDGF-BB] and BDNF). As for GM, *Klebsiella* overgrowth 6 weeks after birth was associated with severe brain injury and inflammatory markers, such as γδ T cells and proinflammatory cytokine secretion, while it was inversely related to neuroprotective secretion.

The relationship between a validated early marker of neurodevelopment, specifically head circumference (HC) growth, and GM establishment from the first week of life was investigated in the prospective study conducted by Oliphant et al. (8). Fecal samples were collected weekly from 58 preterm infants born before 34 weeks of GA during their NICU stay. The poor growth of HC was related to the low abundance of two bacterial taxa that are dominant in adult GMs, Bacteroidetes and *Lachnospiraceae*. Interestingly, the postmenstrual age of 30 weeks was identified as a common timepoint at which both HC growth trajectories and GM composition began to diverge between groups.

Sun et al. (10) characterized the GM of 34 preterm infants in the first month of life during NICU admission and assessed neurodevelopmental outcomes at 36–38 weeks of postmenstrual age or prior to NICU discharge using the Network Neurobehavioral Scale (NNNS) and its Stress/Abstinence subscale (NSTRESS). A functional log-contrast regression model identified GM components at order (Clostridiales, Lactobacillales, Enterobacteriales) and genus (*Veillonella*, *Enteroccoccus*, *Shigella*) level, whose relative abundance variations during the sampling time were associated with the infants’ neurobehavioral outcome as assessed by NSTRESS subscale (10).

The relationship between early GM and neurodevelopment assessed in early childhood was explored in 4 of the studies included in this narrative review. The French national prospective observational cohort study EPIFLORE investigated the association between GM dysbiosis in 577 very preterm infants and long-term outcomes (5). Analysis of GM at 4 weeks after birth identified 6 GM groups influenced by infant characteristics, treatments, and specific NICU clinical strategies, such as ventilation, sedation, feeding, use of antibiotics, and skin-to-skin practice. Notably, after adjustment for confounders, such as GA, absence of assisted ventilation on day 1 was associated with a reduced risk of cluster 5 (driven by *Staphylococcus*) or cluster 6 (including non-amplifiable samples due to low bacterial load), while sedation and low-volume enteral nutrition were associated with increased risk. Skin-to-skin practice was associated with a reduced risk of cluster 5. After adjusting for the above confounder, the authors documented that infants in cluster 4 (driven by *Enterococcus*), 5 and 6 had the highest risk of a 2-year non-optimal outcome, defined as the occurrence of death or neurodevelopmental delay, as assessed by the ASQ at 2 years of age.

In a prospective observational pilot study, we explored the link between GM in the first month of life and neurodevelopment at the correct age of 24 months in 27 very low birth weight (VLBW) infants (6). Neurodevelopmental outcomes, assessed using the revised Griffiths Mental Development Scale (GMDS-R) administered by a psychologist blinded to the GM analysis, were associated with GM features at defined timepoints (taxon abundance) and over time (beta diversity trajectories). Notably, the establishment of GM over time differed based on both the presence and degree of neurodevelopmental impairment. Early GM in neurodevelopmentally impaired infants was rich in *Enterococcaceae* at days 7 and 30, showing a significantly lower abundance of *Bifidobacteriaceae* at day 30 than in neurodevelopmentally normal infants. The abundance of *Bifidobacterium* at 30 days of life was directly related to the GMDS-R General Quotient at 24 months. Neither *Bifidobacterium longum* nor *Bifidobacterium breve* were found in the GM of neurodevelopmentally impaired infants.

The relevance of *Bifidobacterium* in the neurodevelopment of preterm infants was also suggested in the study by Sarkar et al. (7). Stool samples from 24 VLBW infants were collected weekly during their NICU stay, and then at 2 and 4 years of age, to assess the GM establishment in the first years of life. The GM of VLBW infants showed dysbiotic features in the neonatal period, likely related to the NICU environment, and subsequently transitioned to an adult-like GM at 4 years of age. GM features, including diversity and abundance of specific taxa, correlated with several items of the Battelle Development Inventory-2 Screening Test (BDI-2 ST) administered at 2 and 4 years of age. Notably, at 2 years of age, children who did not require neurodevelopmental referral had a *Bifidobacterium*-dominated GM, while *E. coli*, *Citrobacter*, and *Enterobacteriaceae* were highly prevalent in children who required referral. Finally, a randomized clinical trial (11) evaluated neurodevelopmental outcome measured by the Bayley Scales of Infant and Toddler Development (BSID - III) at the corrected age of 2 years in very preterm infants after supplementation with short-chain galacto-oligosaccharides, long-chain fructo-oligosaccharides and pectin-derived acidic oligosaccharides, and possible associations with cytokine levels and stool bacterial counts during the neonatal period. Enteral supplementation with a prebiotic blend during day 3–30 of life did not improve neurodevelopmental outcomes in 77 infants evaluated at 24-month corrected age. However, higher proportions of Bifidobacteria in the GM analyzes at day 7 and day 14 of life were associated with higher BSID Mental Development Index (MDI) scores, while total fecal bacterial counts did not influence the MDI or Psychomotor Development Index (PDI) scores.

## 5. Discussion

In the present narrative review, we considered the existing literature exploring the relationship between early-life GM and neurodevelopment in preterm infants. According to the available evidence, which so far includes only a limited number of clinical studies, monitoring GM dynamics in preterm infants during the first months of life could reveal a possible relationship with later neurodevelopmental outcomes. A relationship has been suggested between both dynamic patterns (i.e., beta diversity trajectories, relative abundance of taxa over time) and static features (i.e., relative taxon abundance or taxonomic clusters at defined timepoints) of GM during the first month of life and brain maturation, as well as neurodevelopmental outcomes in early childhood. Furthermore, some studies have pointed out the potential role of early colonization with specific bacterial taxa, particularly *Bifidobacterium*, on neurodevelopment in early childhood. Specifically, the absence or low relative abundance of *Bifidobacterium* could constitute a biomarker of vulnerability and immaturity, and this observation could potentially lead to early intervention strategies aimed at promoting optimal neurodevelopment in preterm infants during NICU admission and after discharge. Furthermore, *Bifidobacterium* spp. are known to play a pioneering role in the healthy development of the infant GM, contributing to the fine-tuning of the immune system and potentially exerting neuroprotective effects, mainly by modulating the production and release of neuroactive metabolites (83, 84).

However, some limitations of the available evidence need to be recognized. The main limitations relate to the paucity of human studies addressing this topic. Additionally, the small number of subjects included in most published clinical studies has hindered the chance to further explore the impact of various clinical variables (i.e., NEC, LOS, feeding type) on both GM assembly and neurodevelopmental outcome. Another limitation is the time window of GM analysis, as stool samples were mainly collected during the first 30 days of life, and microbial changes after this time window were not investigated. Furthermore, the primary studies were heterogeneous in terms of sample size, clinical evaluations, and methods used to assess neurodevelopmental outcomes. Finally, yet importantly, a major limitation of the GM field is that most studies have focused on the impact of bacterial communities on brain development and subsequent health outcomes in preterm infants, while the potential critical contributions of non-bacterial populations are far from being fully characterized. The importance of considering multi-kingdom interactions when assessing microbiota-mediated effects on human health, particularly in brain development and in the prevention of future neurological disorders, becomes critical as members of microbial communities share the same niches. Consequently, perturbations in one microbial kingdom may also affect the composition and community function of the other kingdoms. Encouraging future studies that delve into this line of research will be essential to realize the full potential of microbiota-targeted solutions to combat the altered microbiota configurations, impaired brain maturation and related health problems that characterize preterm infants.

Evidence from preclinical models has demonstrated that specific bacteria with probiotic properties that confer mental health benefits, also called psychobiotics, can modulate brain function (84, 85). Underlying mechanisms include the production of neuroactive metabolites involved in MGBA, such as gamma-aminobutyric acid and 5-HT, the reduction of proinflammatory cytokines and hypothalamic–pituitary–adrenal activity, as well as GM modulation (86, 87). In the context of the potential psychobiotics effect in early life, it has been suggested that administration of *Lactobacillus acidophilus* and *Bifidobacterium infantis* to pregnant mice promotes brain development and protects the offspring brain from postnatal inflammatory insults (88). More recently, Cowan et al. (89) have demonstrated that early neural maturation in stressed newborn rats was prevented by probiotic administration. Specifically, male Sprague–Dawley rats were reared under standard conditions or exposed to stress induced by maternal separation. The latter animals showed adult-like engagement of the medial prefrontal cortex during fear regulation. However, this response was prevented by the administration of a probiotic blend composed of *Lactobacillus rhamnosus* and *Lactobacillus helveticus*.

Moving from animal model findings to a possible role in humans, prophylactic probiotics have been suggested to reduce the incidence of several clinical outcomes, including NEC, LOS, and mortality in very preterm infants (90), while their potential efficacy as modulators of MGBA and therefore neurodevelopmental outcomes in early childhood is still debated (91, 92). Recent meta-analyzes summarizing the limited literature available on this topic showed no differences in neurodevelopment in infants treated with probiotics or prebiotics compared to controls, while a potential effect of probiotics on short-term growth has been suggested (93, 94).

## 6. Conclusion

Currently available human studies suggest an association between early-life GM, brain development in preterm infants, and neurodevelopmental outcomes. Although a clear mechanistic pathway linking the brain and GM in preterm infants has not yet been elucidated, it could be assumed that specific GM profiles could be the hallmark of neurodevelopmental vulnerability. This observation could pave the way for timely identification of high-risk infants and early intervention strategies aimed at promoting optimal neurodevelopment in preterm infants during the NICU stay and after discharge. Further clinical studies in larger cohorts, possibly integrating multi-omics techniques (e.g., metagenomics, metatranscriptomics, and metabolomics) and animal models, are needed to provide further evidence and mechanistic insights. Besides, studying the MGBA in the context of long-term follow-up of neurodevelopmental outcomes in preterm infants beyond NICU admission is needed to provide insight into potential therapeutic targets and predictive biomarkers for healthy development in preterm infants.

## Author contributions

ST and AA: conceptualization. ST, AA, MB, and IB: methodology. PB, LC, and AS: validation. AA and ST: formal analysis. IB and MB: investigation and writing–original draft preparation. IB: resources and data curation. ST, AA, PB, LC, and AS: writing–review and editing. All authors have read and agreed to the published version of the manuscript.

## Conflict of interest

The authors declare that the research was conducted in the absence of any commercial or financial relationships that could be construed as a potential conflict of interest.

## Publisher’s note

All claims expressed in this article are solely those of the authors and do not necessarily represent those of their affiliated organizations, or those of the publisher, the editors and the reviewers. Any product that may be evaluated in this article, or claim that may be made by its manufacturer, is not guaranteed or endorsed by the publisher.
